# Disturbances in the ploidy level in the gynogenetic sterlet *Acipenser ruthenus*

**DOI:** 10.1007/s13353-017-0389-2

**Published:** 2017-02-06

**Authors:** D. Fopp-Bayat, K. Ocalewicz, M. Kucinski, M. Jankun, B. Laczynska

**Affiliations:** 1 Department of Ichthyology, University of Warmia and Mazury in Olsztyn, 10-719 Olsztyn, Poland; 2 Department of Ichthyology, University of Warmia and Mazury in Olsztyn, Oczapowskiego 5, 10-956 Olsztyn, Poland; 3 Department of Marine Biology and Ecology, Institute of Oceanography, University of Gdansk, 81-378 Gdynia, Poland

**Keywords:** *Acipenser ruthenus*, Albino form, Colour body marker, Genetic verification, Gynogenetic progeny, Mitotic gynogenesis

## Abstract

Artificial mitotic gynogenesis, a chromosome set manipulation, is applied to provide the homozygous progeny with only maternal inheritance. Here, gynogenetic development was induced in the sterlet *Acipenser ruthenus* L. (Acipenseridae) by activation of the eggs originating from albino females with the UV-irradiated spermatozoa from wild-coloured males, followed by the heat shock applied to suppress the first mitotic division in the haploid zygotes. All experimentally obtained gynogenetic offspring possessed recessive albino coloration. Moreover, the genetic verification, based on three microsatellite DNA markers, confirmed the only maternal inheritance in the albino progeny. Cytogenetic screening enabled identification of the aneuploids, haploids, diploids, triploids, tetraploids and mosaic individuals among the gynogenetic larvae that hatched from the eggs subjected to the heat shock. Furthermore, 40% of the larvae from the haploid variants of the research that were not exposed to the temperature shock showed the diploid chromosome number. A variation of the ploidy level observed in the gynogenetic sterlets may be the consequence of the spontaneous polyploidisation that occurred in the haploid zygotes. Moreover, observation during embryogenesis showed varied stages of eggs development and the asynchronous cell cleavages that may have resulted in the chromosomal disturbances observed in the gynogenetic sterlets here.

## Introduction

Genome manipulations such as gynogenesis and androgenesis are techniques leading to the production of individuals possessing only maternal or only paternal chromosomes, respectively. Application of the motile spermatozoa with UV-inactivated nuclear genome for the eggs activation results in the development of haploid gynogenetic individuals. In turn, activation of the irradiated eggs performed by the untreated sperm allows for induction of the androgenetic development. Further exposition of the gynogenetic and androgenetic haploid zygotes for the temperature or pressure shock inhibiting release of the second polar body (meiotic gynogenesis) or suppressing the first mitotic cleavage (mitotic gynogenesis and androgenesis) enables recovery of the diploid state and production of the heterozygous gynogenotes or gynogenetic and androgenetic doubled haploids (DHs), respectively (Arai 2001). Gynogenesis and androgenesis have been applied in research concerning sex determination systems in fish and providing all female or all male stocks. Moreover, mitotic gynogenesis and androgenesis provide fully homozygous fish that may be applied in fish breeding programmes and studies concerning the role of recessive alleles during fish ontogeny (Devlin and Nagahama 2002; Komen and Thorgaard 2007).

Verification of the genome manipulations efficiency is as important as induction of these processes. Only maternal or only paternal chromosomes inheritance may be analysed at three different levels. The efficacy of the radiation-induced genetic inactivation of the eggs (androgenesis) or spermatozoa (gynogenesis) may be verified easily by application of the gametes originating from albino males or albino females, respectively, as albinism is usually a recessive trait (Ocalewicz et al. 2004; Fopp-Bayat and Ocalewicz 2015). Recovery of the diploid state in fish from the androgenetic and gynogenetic experiments must also be confirmed; thus, such studies require the application of tools to confirm the ploidy level of the provided progeny (Fopp-Bayat and Woźnicki 2006). Thirdly, uniparental nuclear DNA inheritance and homozygosity must be approved, which is usually achieved in the course of the microsatellite analysis performed in the gynogenetic and androgenetic offspring, as well as on egg and sperm donors (Fopp-Bayat 2007a, b; Fopp-Bayat and Woznicki 2007).

To date, chromosome set manipulation has been implemented in the breeding programmes of several aquaculture species, including salmonids, cyprinids, flatfishes and sturgeons (Purdom 1993). Unfortunately, results concerning the production of DHs in sturgeons have not been published to date, which is surprising taking into account that sturgeon aquaculture is a fast developing sector of fish production. Because sturgeons are sexually monomorphic without any external sexual characteristics, the application of innovative methods for the production of female lines is very important in their aquaculture. In sturgeons, females are heterogametic sex (WZ genotype) and gynogenesis can be induced to generate ‘super-females’ (WW genotype) that, when subsequently crossed with normal males (ZZ genotype), give all-female offspring and have been induced in several sturgeon species (Van Eenennaam et al. 1996; Flynn et al. 2006; Fopp-Bayat 2007a, b). However, to date, the only gynogenetic experiments conducted in sturgeons were of the meiotic type. Gynogenetic offspring produced with the application of early shock consisted of males (ZZ) and females showing both homogamety (WW) and heterogamety (WZ). The application of late shock would allow to produce males and only homogametic females (WW). Thus, the main goal of this research was to induce mitotic gynogenetic development and produce DHs. The research was performed using sterlet *Acipenser ruthenus* albino females and wild-coloured males. Sterlet is the smallest, freshwater Acipenseridae species. Under aquaculture conditions, sterlet reaches sexual maturity at around 3 to 6 years old, which is the earliest among all the sturgeons. Easy access and early maturation make sterlet a useful model in sturgeon reproduction research, including genome manipulations and interspecies transfer of primordial germ cells, leading to the generation of germline chimeras (Pšenička et al. 2011). Eggs obtained from albino females have been activated by UV-inactivated spermatozoa provided from the wild-coloured males. Survival rates of the gynogenetic embryos were monitored until hatching. Molecular and cytogenetic analyses were applied to verify only maternal inheritance in the genome-manipulated offspring and to check their ploidy, respectively.

## Materials and methods

### Experimental design

This study was carried out in strict accordance with the recommendations in the Polish ACT of 21 January 2005 of Animal Experiments, Dz. U. z 2005 r. Nr 33, poz. 289. The protocol was approved by the Local Ethical Committee for Experiments on Animals of the University of Warmia and Mazury in Olsztyn, Poland (permit number: 75/2012).

Gamete donors for this study were derived from the sturgeon broodstocks maintained in Wasosze fish farm, Poland. Four albino sterlet females aged 6 years with an average body weight of 3000 g (Female A, Female B, Female C and Female D) and four wild-coloured sterlet males aged 5 years with an average body weight of 3000 g (Male A, Male B, Male C and Male D) were used to provide eggs and spermatozoa in order to induce gynogenesis. Before controlled reproduction, all spawners were genotyped using three microsatellite DNA loci (*Afu-68*, *AfuB-68* and *Spl-163*). The ‘controlled pairing’ of spawners was applied based on differences in genetic profiles among females and males (Kaczmarczyk and Fopp-Bayat 2013). The collection of gametes and assessment of their quality were performed as previously described (Fopp-Bayat et al. 2007). One sterlet female produced about 250 to 300 g of the ovulated eggs. Approximately 50 mL of sperm from each sterlet male was obtained during a single suction performed using a syringe. Milt from Male A and Male C was centrifuged and its seminal fluid was applied for dilution of the Male B, Male C and Male D spermatozoa. Diluted sperm (1:9) was exposed to UV radiation at a dose of 135 J/m^−2^ (Fopp-Bayat et al. 2007).

Data published by Dettlaff and Vassetzky (1991) and our observations made on the sterlet embryos at the two-blastomere stage indicated that the first mitotic division occurred at a temperature of 15 °C, usually between 160 and 180 min post-activation (mpa). However, eggs with early or delayed first cell cleavage were observed in the same batch of eggs as well. In the present study, two experiments were performed to investigate the efficiency of the temperature shock applied at 160, 170 and 180 mpa to disturb the first cell cleavage in the sterlet eggs.

**Fig. 1:**
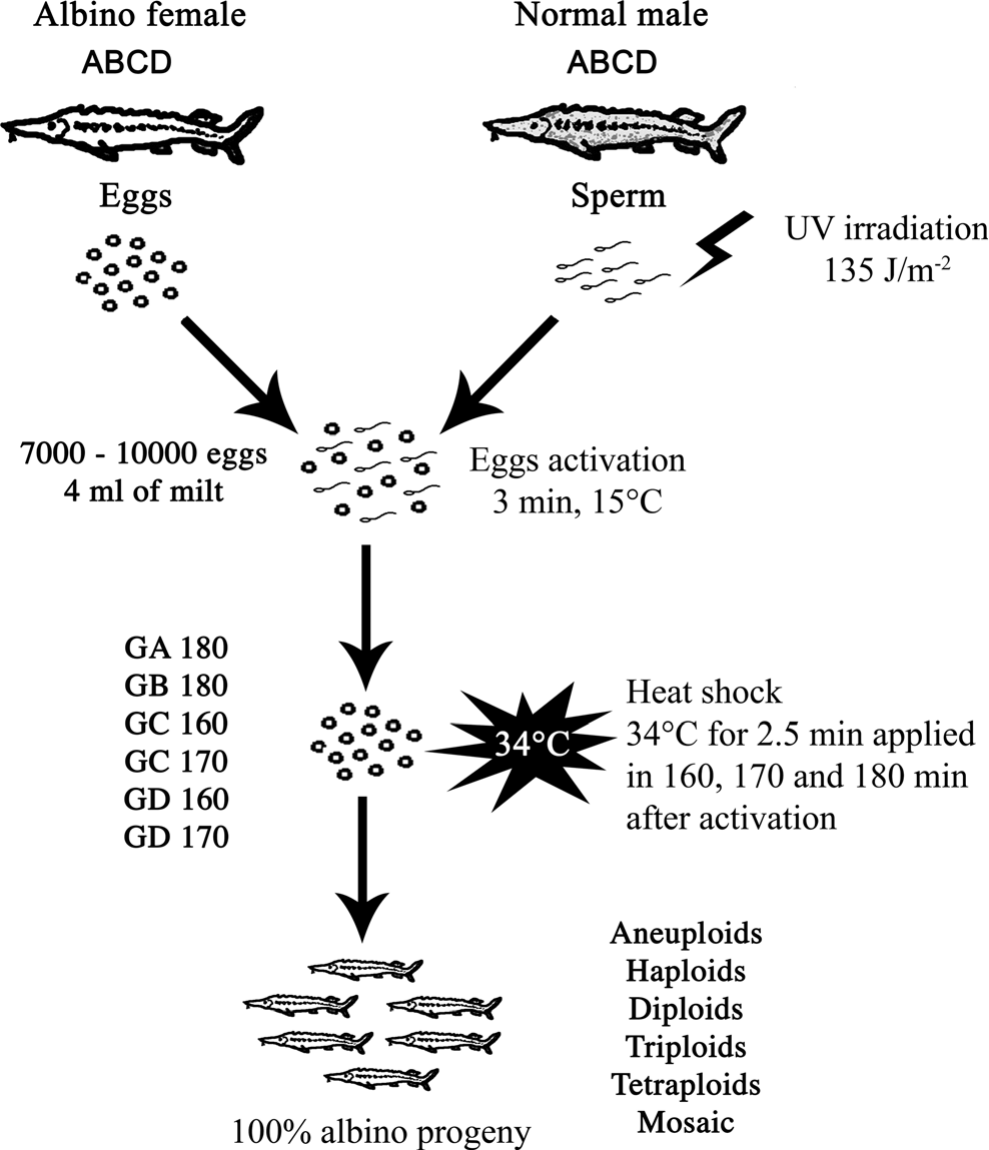
Scheme of the gynogenetic experiments in sterlet *Acipenser ruthenus*

In Experiment I, albino sterlet eggs from Female A (20,000 eggs) and Female B (20,000 eggs) were activated with the irradiated sterlet spermatozoa (Male B). The egg desticking was conducted using NaCl-tannic acid solutions (Feledi et al. 2011). In the diploid gynogenetic variants of the experiments (GA 180 and GB 180), part of the activated eggs (10,000 eggs from each female) were exposed to heat shock (34 °C for 2.5 min applied at 180 mpa) to suppress the first cell cleavage and to restore diploid state in the gynogenetic zygotes (Fig. 1). Eggs collected from Female A and Female B that were not subjected to the heat shock constituted the haploid control groups (HA and HB, respectively). Additionally, ova quality was checked by eggs fertilisation (10,000 eggs from each female) with the non-irradiated sterlet sperm (CA and CB groups).

Another experiment (Experiment II) was conducted with the albino sterlet eggs from Female C (21,000 eggs) and Female D (21,000 eggs) and the UV-irradiated sperm of wild-coloured sterlet. To suppress the first mitotic division and to restore diploid state in the mitotic gynogenetic zygotes, part of the activated eggs (14,000 eggs divided into two groups) were exposed to a heat shock of 34 °C for 2.5 min applied 160 (GC 160 and GD 160) and 170 (GC 170 and GD 170) mpa (Fig. 1). The haploid (HC and HD) and normal diploid (CC and CD) control groups were also provided in this experiment (7000 eggs in each experimental group).

In both experiments, an incubation temperature of 15 °C was maintained between eggs activation and exposure to the heat shock. To avoid photoreactivation, the sperm and embryos were stored in the absence of light for 5 h. Survival rates of embryos and larvae were recorded at different developmental stages: fertilisation (3 h post-activation, hpa), neurulation (50–60 hpa) and hatching (5–6 days post activation, dpa). Unfertilised eggs and dead embryos were removed to prevent fungal infection. At 5–6 dpa, the number of free-swimming larvae was counted in each treatment. Abnormal larvae and unhatched eggs were removed from the tank and also counted. Hatched larvae from each of the individual treatments were transferred from the hatching system to a rearing system with flowthrough aerated water (16 °C), where they remained until sampling for the molecular and cytogenetic analysis.

### Genetic verification of gynogenetic progeny

Randomly selected larvae (*n* = 15–35) from the experimental groups and controls were sampled 6 days post-hatching (dph) for cytogenetic and molecular analyses. The tail from each sampled larvae and fin clips from the four albino females were stored in 96% ethanol for the molecular analysis. The 0.5-mL sample of sperm from each male was frozen and stored at −70 °C for the DNA isolation. Genomic DNA for the amplification of three microsatellite loci, *Afu-68*, *AfuB-68* and *Spl-163* (May et al. 1997; McQuown et al. 2000), were isolated from tails and fin clips using the Sherlock DNA purification kit (A&A Biotechnology, Poland), according to the manufacturer’s procedure. Genomic DNA isolation from spermatozoa was conducted according to the procedure described by Fopp-Bayat and Ciereszko (2012). Reaction mixes were prepared in a total volume of 25 μL with 40 ng of DNA template, 1× polymerase chain reaction (PCR) reaction buffer (50 mM KCl, pH 8.5; Triton X-100), 0.4 mM of each primer, 0.25 mM of each deoxynucleotide triphosphate (dNTP), 3.3 mM MgCl_2_ and 0.6 units of GoTaq Flexi DNA Polymerase (Promega, Madison, WI, USA). Re-distilled water was used to bring the reaction mixture to the desired final volume. Amplification was conducted with a MasterCycler Gradient Thermal Cycler (Eppendorf, Germany), with initial denaturation at 94 °C for 5 min, followed by 33 amplification cycles (94 °C, 1 min; 53–55 °C, 30 s; 72 °C, 30 s) and final elongation at 72 °C for 5 min. In order to enable genotyping of PCR products with an Applied Biosystems 3130 Genetic Analyzer, forward primers were 5′-labelled with the different fluorescent reporter dyes (*Afu-68*-FAM, *AfuB-68*-VIC, *Spl-163*-NED). The lengths of the amplified DNA fragments were determined using an Applied Biosystems 3130 Genetic Analyzer sequencer against GeneScan 600 LIZ dye Size Standard (Applied Biosystems, Foster City, CA, USA). Fragment sizes and alleles were determined using the GeneMapper and Genetic Analyzer software (Applied Biosystems, Foster City, CA, USA), according to the manufacturer’s recommendations.

The same microsatellite DNA markers were initially analysed in the parental individuals. Discrimination of the fingerprint pattern was based on the evidence of female-specific bands, which were present in the gynogenetic progeny in each of the experimental groups.

### Cytogenetic verification of experimental progeny

Larvae from the gynogenetic and control groups were placed in 0.025% solution of colchicine for 4 h at room temperature. After incubation, larvae were sacrificed and their heads placed in cold hypotonic solution (0.075 M KCl) and incubated for 1 h at 4 °C. After the treatment with KCl solution, a few drops of freshly prepared fixative (methanol:acetic acid, 3:1) were added and the samples were stored in the refrigerator for 15 min. Next, tissue samples were placed in the freshly prepared fixative, which was changed 2–3 times afterwards. Finally, hypotonised and fixed tissues were homogenised by preparation needles in the presence of the fixative. One or two drops of the provided cell suspensions were placed on microscope slides and left to dry. Afterwards, metaphase spreads were stained with Giemsa staining. Between two and six high-quality metaphase spreads from each cytogenetically studied larvae were analysed under a Zeiss Axio Imager A1 microscope equipped with a fluorescent lamp and a digital camera. Captured images were electronically processed using Band View/FISH View software (Applied Spectral Imaging).

## Results

**Fig. 2:**
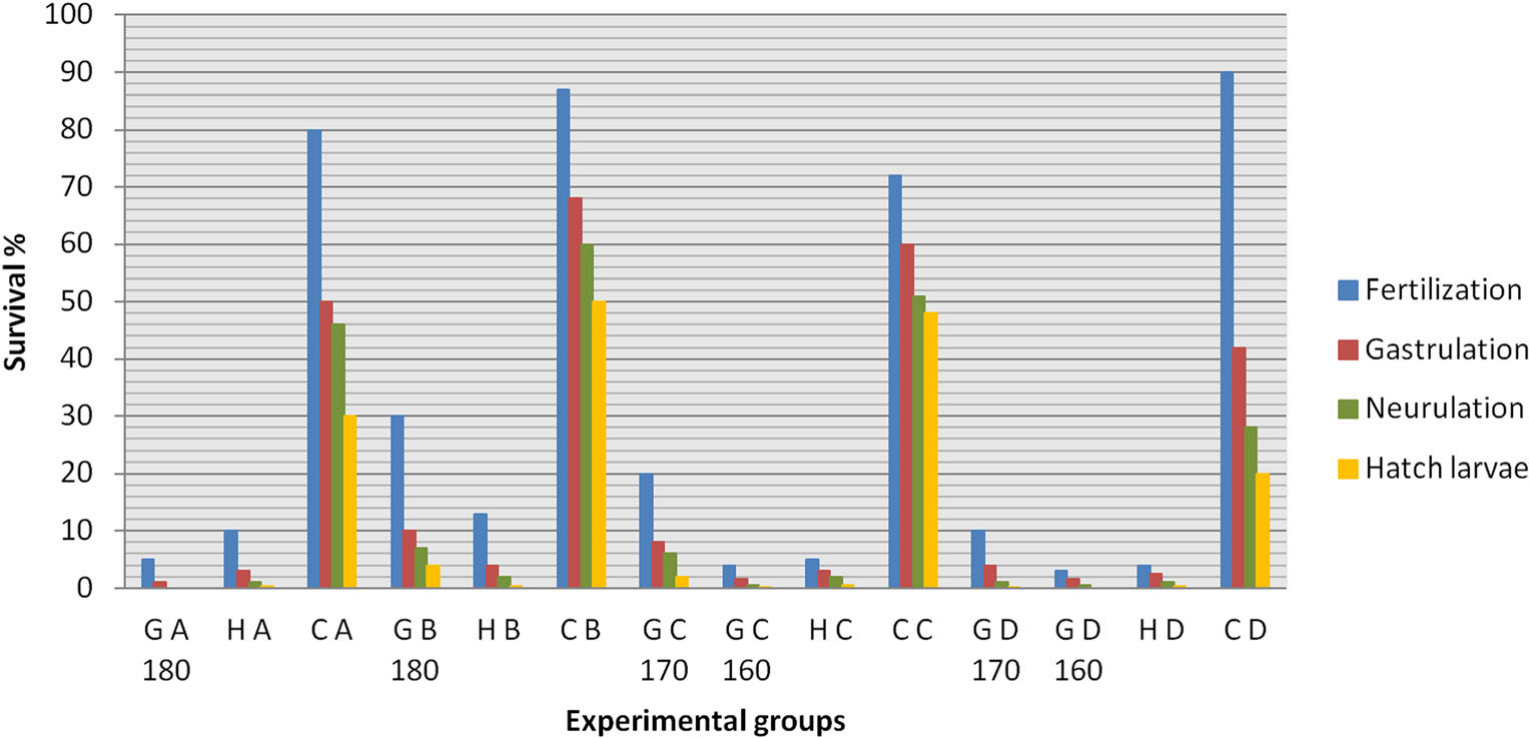
Survival rate (%) of the experimental manipulated groups of sterlet *Acipenser ruthenus* during embryogenesis. Y-axis: survival rates (%); X-axis: experimental groups

**Fig. 3:**
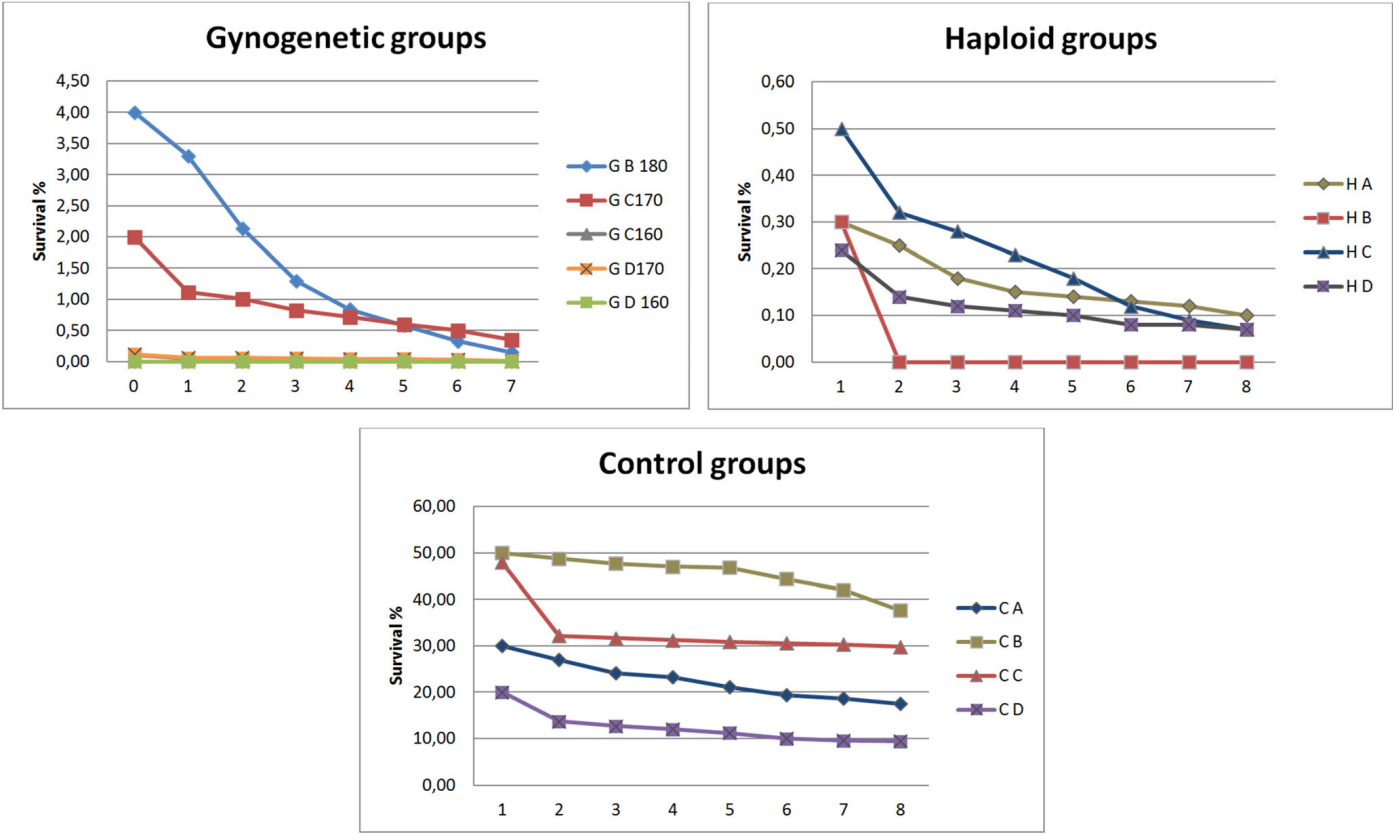
Survival rate (%) of the experimental (haploid, gynogenetic and control) groups of sterlet *Acipenser ruthenus*. Y-axis: survival rates; X-axis: days post-hatching

Application of the UV-irradiated sperm derived from wild-coloured sterlet for activation of the albino sterlet eggs, followed by heat shock at 34 °C in the 160th, 170th and 180th minutes was successful and resulted in production of the viable putative gynogenetic progeny that hatched in four experimental groups (GB 180, GC 170, GC 160, GD 170). In the GA 180 and GD 160 groups, all embryos died during the early stages of onthogenesis. In the experimental haploid groups (HA, HB, HC and HD), about 0.25–0.5% living hatched larvae were also observed (Fig. 2). The ‘colour marker’ inherited from the albino mothers was identified in all the progeny from the experimental gynogenetic groups. The survival of fish in the gynogenetic and control groups to hatching is summarised in Fig. 2. The survival of the hatched larvae within the first several days of rearing in the gynogenetic and control groups are compared in Fig. 3. More than 90% of the embryos in the gynogenetic haploid and gynogenetic diploid groups died at an early stage of embryonic development. Moreover, different abnormalities in the embryos were observed. In the hatched larvae, body malformations including distortion of the body, tail deformation, underdevelopment of the head, reduced yolk sac etc. were observed.

All breeders (four females and four males) in each group were initially identified by diagnostic markers and the specific profiles, based on three microsatellite DNA loci, were provided. Paternity test, based on the microsatellite DNA markers applied in all experimental groups of sterlet, revealed the only maternal inheritance.

The results of the microsatellite DNA analysis showed that there was no genetic contribution from the paternal genome in the fish from the gynogenetic groups (Table 1).

**Table 1:** Observed genotypes at microsatellite loci: *Afu-68*, *AfuB-68* and *Spl-163* in gynogenetic progeny of sterlet *Acipenser ruthenus* L.

Sample, locus	Observed genotypes		
	*Afu-68*	*AfuB-68*	*Spl-163*
Female B	200/200	110/118/178	186/194
Male B	108/208	158/186	200/200
GB 180 (*n* = 15)	200/200	110/118/178	186/194
		110/118	194/194
Female C	180/200	110/158/178	186/186
Male C	204/204	118/186	194/200
GC 160 (*n* = 5)	180/180	110/178	186/186
	180/200	158/178	
	200/200		
GC 170 (*n* = 35)	180/180	110/158	186/186
	180/200	110/158	
	200/200	158/158	
		158/178	
		110/158/178	
Female D	180/204	110/118/158	186/200
Male D	200/200	118/178	194/194
GD 170 (*n* = 5)	180/180	110/118	186/186
	180/204	110/158	186/200
		110/118/158	

GB, GC, GD: gynogenetic progeny of females B, C and D, respectively

**Fig. 4:**
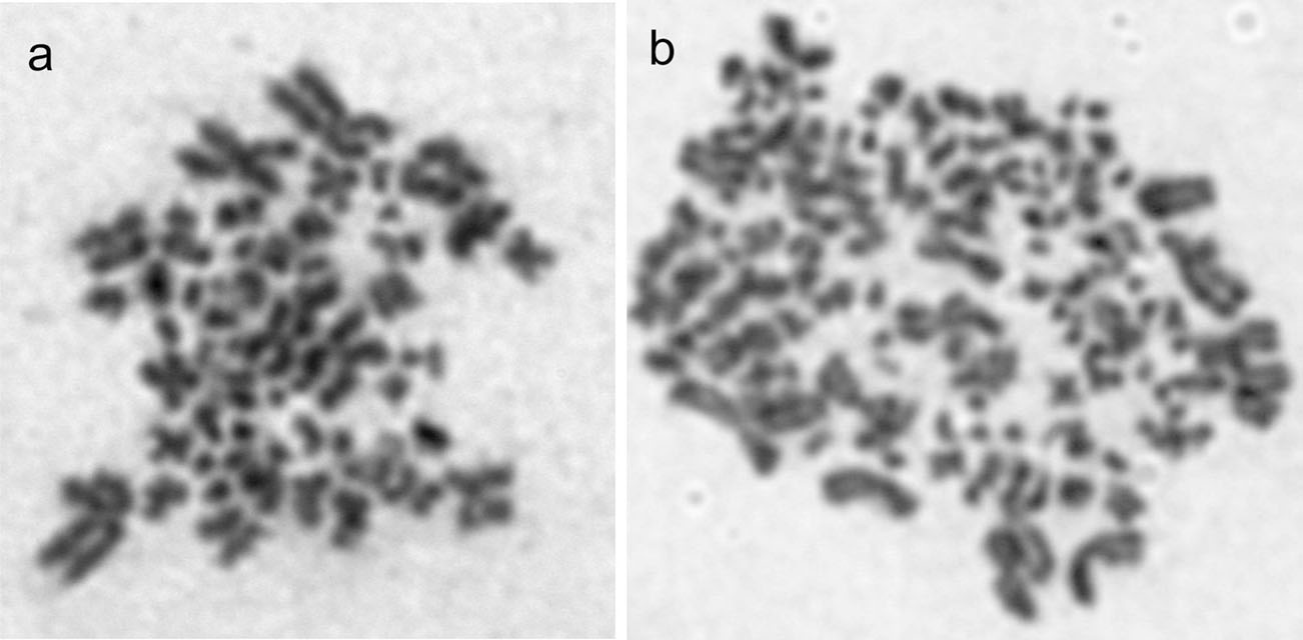
Metaphase spreads of haploid (1n = 60) (**a**) and spontaneous diploid (2n = 120) (**b**) gynogenetic sterlets *Acipenser ruthenus*

**Fig. 5:**
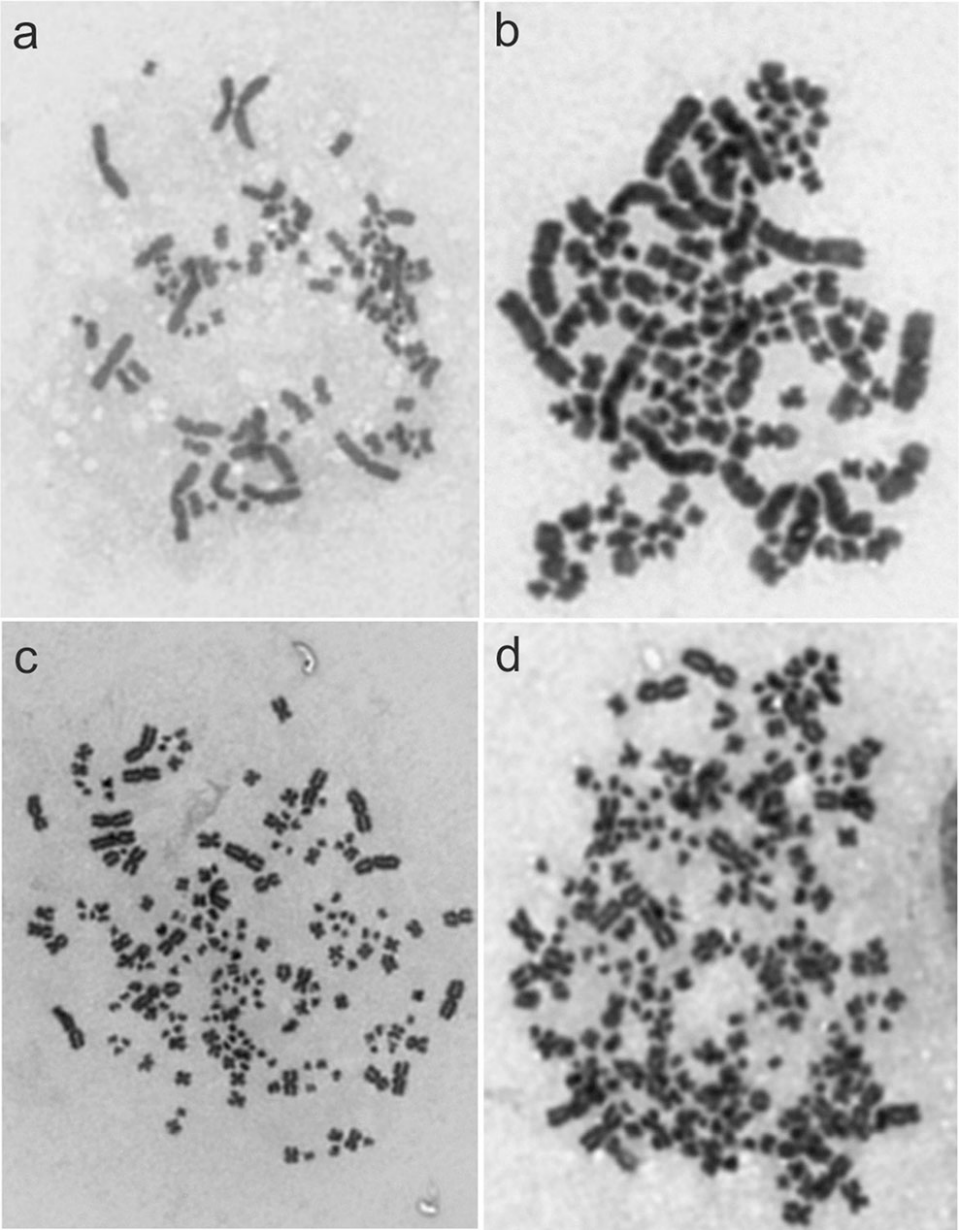
Metaphase spreads of aneuploid (90) (**a**), diploid (2n = 120) (**b**), triploid (180) (**c**) and tetraploid (4n = 240) (**d**) gynogenetic sterlets *Acipenser ruthenus*

Cytogenetic analysis of the gynogenetic larvae from the haploid and diploid variants of Experiment I exhibited the presence of inter- and intraindividual variations in the chromosome numbers (Figs. 4 and 5). Haploid (33.3%), diploid (40%), aneuploid and mosaic (26.7%) individuals were found within the larvae from the haploid variant of the experiment. Among larvae that hatched from the eggs subjected to temperature shock, individuals with chromosome number equalling ≈240 (20%), ≈180 (10%) and ≈90 (40%) were observed (Fig. 5). Moreover, in some individuals (30%), intercellular variation of the chromosome numbers was observed. Such mosaic larvae consisted of a mixture of haploid (60), diploid (120) and triploid (180) cells.

In haploid variants from Experiment II, few of the gynogenotes showed doubled chromosome sets. In turn, in the groups treated with heat shock, individuals with ≈120 chromosomes (20%), 60 chromosomes (20%), aneuploids (>60 chromosomes) (40%) and mosaics (20%) were reported.

## Discussion

Sturgeons have evolved about 200 million years ago and several genome duplication events followed by speciation have resulted in 27 extant sturgeon species. Based on the chromosomes number, sturgeons are divided into three groups: group A with about 120 chromosomes, group B with 250–270 chromosomes and group C with only one species, the shortnose sturgeon (*Acipenser brevirostrum*), which have 360 chromosomes (Fontana et al. 2001, 2003; Havelka et al. 2013a, b). At present, incidences of spontaneous polyploidisations have also been described in several sturgeon species. Spontaneous triploids were exhibited among progenies of bester (*Huso huso* × *Acipenser ruthenus*), white sturgeon (*A. transmontanus*), Siberian sturgeon (*A. baerii*), *Huso dauricus*, sterlet and Sakhalin sturgeon (*A. mikadoi*) (Omoto et al. 2005; Zhou et al. 2011, 2013; Havelka et al. 2013b; Schreier et al. 2013). In the latter species, some portion of the tetraploid specimens was also obtained. Polyspermy (1), pre-meiotic endomitosis leading to chromosome doubling and the production of unreduced gametes (2) and inhibition of extrusion of the second polar body after fertilisation (3) may be considered as the most probable mechanisms responsible for the spontaneous polyploidisation in sturgeons. Unfortunately, the phenomenon of spontaneous polyploidisation in sturgeons interfered with the process of artificial induction of DH development performed in the present study.

In sturgeons, studies on the chromosome set manipulations have some limitations. Firstly, the number of sturgeon spawners is small (especially albino specimens) and the fish reach sexual maturity relatively late. Secondly, the survival of sturgeon individuals produced within chromosome sex manipulations is extremely low and to obtain viable larvae is often impossible. Thus, the number of eggs used for the gynogenetic experiments should be as high as possible. Finally, sterlets produce a small number of eggs, which precludes to design experiments containing numerous and large groups and variants. Therefore, to increase the chance of producing living gynogenetic offspring in Experiment I, all eggs obtained from each female (Female A and Female B) were divided into only three parts (GA 180, HA, CA and GB 180, HB, CB, respectively). Cytogenetic analysis of the gynogenetic larvae from Experiment I revealed that the thermal shock might have been applied too late (180 mpa). Therefore, the other experiment (Experiment II) with temperature shocks applied earlier (160 and 170 mpa) was performed. In this experiment, the eggs obtained from each female were divided into four batches (GC 170, GC 160, HC, CC and GD 170, GD 160, HD, CD, respectively). Some of the gynogenetic larvae from the haploid experimental variants exhibited diploid chromosome number. Moreover, among gynogenotes hatched from eggs exposed to heat shock, individuals with unexpectedly high chromosome numbers were also observed. However, all larvae hatched from eggs activated by the irradiated spermatozoa were shown to have albino coloration and possess only maternal microsatellite DNA markers. Firstly, this proved that radiation-induced inactivation of the paternal chromosomes was successfully performed. Secondly, it suggested that disturbances in the ploidy level among gynogenetic sterlets were caused by maternal effects, including endomitosis or retention of the second polar body. Incidences of spontaneous polyploidisations usually take place when poor quality ova, namely, over-ripened and aged eggs, are used for fertilisation. The post-ovulatory aging leading to spontaneous polyploidisation have been reported in many cultured fish species (Yamazaki et al. 1989; Aegerter and Jalabert 2004; Flajšhans et al. 2007; Ocalewicz and Dobosz 2009, among others). The application of heat shock to unreduced eggs must have resulted in the production of gynogenetic larvae with 240 chromosomes observed in Experiment I. In turn, larvae with 180 chromosomes could be triploids with genomes composed of two sets of maternal chromosomes and one set of paternal chromosomes that was not efficiently inactivated by UV light. On the other hand, such individuals may have also appeared in the course of retention of the second polar body and duplication of the maternal chromosomes from the maternal pronucleus.

Other chromosomal disturbances observed among gynogenetic sterlets from the groups subjected to heat shock may be related to the asynchronous cell cleavages in the gynogenetic haploid sterlet zygotes. The preliminary observations of early embryological development exhibited by sterlet eggs used in the present research were at varied stages of maturation and showed different timing of the first mitotic division. Thus, it is reasonable to assume that the heat shock applied to the batch of fertilised eggs may well inhibit release of the second polar body in some of the eggs, disturb further stages of zygote development in others or it may be completely inefficient missing both meiosis and the first cell cleavage. The occurrence of haploid larvae among specimens hatched from the eggs exposed to heat shock confirmed the last assumption.

Observations of the aneuploids and mosaics among gynogenetic sterlets may also be explained by post-ovulatory aging of the eggs. Over-ripening of ova has been proposed to be responsible for the occurrence of aneuploids and mosaics among Japanese eels (*Anguilla japonica*) (Nomura et al. 2013). Moreover, it has been proposed that a shock applied to restore the diploid state in the zygotes of the genome-manipulated fish does not impair the first cell cleavage but the second one, which, in turn, may lead to the formation of haploid/diploid mosaic individuals (Zhang and Onozato 2004). Mosaics and aneuploids have also been observed among androgenetic and gynogenetic DHs in the salmonid fish species (Tanaka et al. 2003; Ocalewicz et al. 2010; Michalik et al. 2015).

## Conclusions

In the present research, induced gynogenetic sterlets exhibited large ploidy variation. As all individuals hatched from eggs activated by UV-irradiated spermatozoa were proved to have only maternal DNA, it has been assumed that disturbances in the ploidy level were caused by maternal effects, including endomitosis or retention of the second polar body. Moreover, the appearance of aneuploids and mosaic individuals among gynogenetic specimens suggested that sterlet eggs used in the experiments were at different stages of development or had decreased quality related to post-ovulatory aging. The asynchronous cell cleavages in the gynogenetic zygotes precluded efficient application of heat shock to suppress the first mitotic cleavage and production of doubled haploids (DHs).
